# Diverse ossification pathways in the lower jaw of *Sclerocephalus* (Lower Permian) as revealed by the first record of chondroid bone in Amphibia

**DOI:** 10.1038/s41598-026-68964-y

**Published:** 2026-09-15

**Authors:** Dorota Konietzko-Meier, Nicole Klein

**Affiliations:** 1 Staatliches Museum für Naturkunde Stuttgart, Rosenstein 1, 70191 Stuttgart, Germany; 2 Bonner Institut für organismische Biologie, Paläontologie [BIOB-V], Universität Bonn, Nussallee 8, 53115 Bonn, Germany

**Keywords:** Temnospondyli, Early ontogeny matrices, Ossification strategies, Rapid growth, Dermal bones, Meckelian cartilage, Anatomy, Developmental biology, Evolution, Zoology

## Abstract

Dermal bones are typically thought to form directly from mesenchyme, however some can develop via a transient tissue called chondroid bone, which are well documented among fishes and amniotes. Here, we report the first occurrence of chondroid bone among non-amniote tetrapods and the stratigraphically oldest record of this tissue among vertebrates, in the temnospondyl *Sclerocephalus nobilis* from the Lower Permian of Germany. Histological analysis of its mandible reveals a heterogeneity of developmental pathways: the angular forms through direct intramembranous ossification, the articular ossifies endochondrally from Meckelian cartilage, and the dermal surangular uniquely contains chondroid bone, with chondrocyte-like cells embedded in the matrix composed of interwoven loop complexes. This tissue likely represents an early developmental stage, promoting rapid expansion of the bone surface while potentially providing mechanical support in regions experiencing high stress during feeding. The angular exhibits a diploë structure and is composed of lamellar and parallel-fibred bone, with remnants of avascular faint parallel-fibred bone representing the larval stage. The articular represents the only endochondral bone and consists of an early-formed periosteal bone that is parallel-fibred in nature, with prominent Sharpey’s fibers and a secondary trabecular endosteal region. The discovery of chondroid bone in *Sclerocephalus* expands the known diversity of skeletal tissues among early tetrapods and suggests that transitional bone types may have been more widespread than previously recognized, but are likely underreported due to preservational biases and limited sampling.

## Introduction

First described as “Knorpel Knochen” by Schaffer^1^, chondroid bone is a primary, transitional skeletal tissue that combines features of both cartilage and bone, consisting of chondrocyte-like cells embedded in a partially mineralized matrix^2–5^. Traditionally the chondroid bone is separated into type I, related with dermal bones and type II—typical for endochondral bones^2^. The type II of chondroid bone is related to endochondral bone formation and results from the incomplete ossification of the cartilage template^2^. For a long time, intramembranous (dermal) bones were assumed to follow a single developmental pathway via direct ossification from mesenchyme without an intermediate cartilage stage^6^. Later studies, however, have shown that dermal bones may as well develop through a transient cartilage-like stage, in which the anlage consists of the chondroid bone type I^2–4^.

In extant taxa, chondroid bone is identified histologically using stained decalcified sections; however, this approach is not applicable to fossil material^2–4^. Consequently, the recognition of chondroid bone in fossils is challenging due to the rarity of this tissue, its limited study in paleohistology, and the lack of consistent diagnostic criteria. In petrographic thin sections, chondroid bone can be distinguished from the surrounding bone matrix by a combination of histological features. It is characterized by large, rounded to irregular cell lacunae resembling hypertrophic chondrocytes and clearly distinct from osteocyte lacunae, which are typically smaller, more elongate, and surrounded by a network of canaliculi. Chondroid bone also exhibits a higher cell density than the adjacent bone, and a matrix that differs from the surrounding bone and may superficially resemble diagenetically altered bone [^4^ and references therein].

In teleost fishes, chondroid bone is a relatively common component of dermal cranial elements, especially the opercular series, as well as other dermal bones of neurocranium and jaws, particularly near articular surfaces. It is associated with early ontogenetic rapid growth, supports areas exposed to high stress from feeding and movement and forms as a regenerative tissue^5,7–11^. In contrast, chondroid bone in extant and fossil amniotes is much less frequently reported as for fishes, but when present it occurs in association with both dermal and endochondral ossification [^3,12–18^ and references therein]. Among amniotes, chondroid bone appears to serve primarily a developmental role, reflecting early ontogenetic adaptations associated with rapid growth and sutural expansion of intramembranous bones, but it rarely has been observed as regenerative tissue^3,4,15,19–24^.

**Fig. 1:**
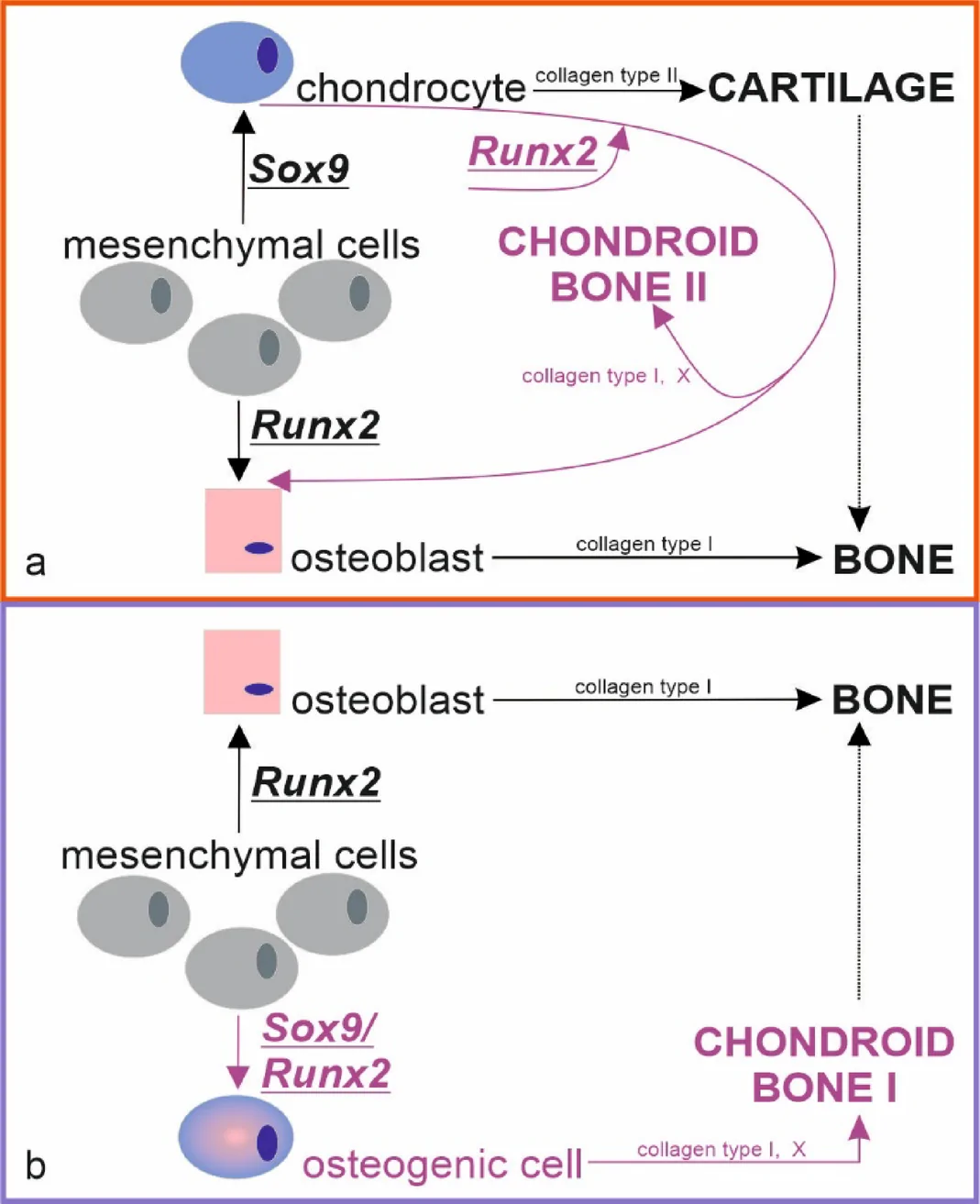
Schematic overview of chondroid bone development during (**a**) endochondral and (**b**) intramembranous ossification. (**a**) During endochondral ossification, mesenchymal cells first differentiate into SOX9-regulated chondroblasts, forming the cartilage template. Subsequently, osteogenesis is initiated by RUNX2-regulated osteoblast precursors, while some hypertrophic chondrocytes may transdifferentiate into osteoblasts, contributing primarily to the formation of chondroid bone type II. (**b**) During intramembranous ossification, mesenchymal cells differentiate directly into RUNX2-regulated osteoblasts. However, these mesenchymal cells retain the capacity for SOX9-mediated chondrogenesis. The simultaneous activation of osteogenic and chondrogenic programs results in the formation of chondroid bone type I (summarized after [^24^ and references therein]).

The formation of chondroid bone results from the developmental trajectory of mesenchymal stem cells (Fig. 1). The precursors of the skeletal cells arise from two embryonic structures, mesoderm (paraxial and lateral plate) and cranial neural crest, both forming the endochondral and dermal bones^24^. Mesodermal mesenchyme contributes primarily to the axial and appendicular skeleton, including vertebrae, ribs, limb elements, and parts of the chondrocranium, whereas cranial neural crest-derived mesenchyme generates much of the splanchnocranium skeleton, including Meckelian cartilage^24^.

Typically, skeletal elements are formed by only two cell types: chondrocytes, responsible for cartilage production and generally restricted to endochondral ossification, and osteoblasts, located primarily in the periosteum, which deposit bone matrix in both endochondral and dermal bones^24–30^. During embryogenesis, interacting gene networks regulate the differentiation of progenitor cells into either chondrocytes or osteoblasts, depending on the expression of specific transcription factors, RUNX2 promoting osteoblast differentiation, whereas SOX9 is required for chondrocyte development^24^.

During endochondral ossification, mesenchymal cells first condense and differentiate into chondroblasts under the control of SOX9, forming the cartilage template (Fig. 1a). Osteogenesis is subsequently initiated by a following population of RUNX2-controled mesenchymal progenitors, which differentiate into osteoblasts (Fig. 1a). In addition, some hypertrophic chondrocytes may transdifferentiate into osteoblasts (Fig. 1a), a process that contributes mostly to the formation of chondroid bone type II^24^.

In contrast, dermal bones develop via intramembranous ossification, in which mesenchymal cells, independent from the embryological origin, differentiate directly into osteoblasts under the control of RUNX2 (Fig. 1b). However, the mesenchymal precursors of dermal bones, particularly those derived from cranial neural crest, retain the potential for SOX9-driven chondrogenesis. These cells can simultaneously activate osteogenic and chondrogenic programs, producing chondroid bone (Fig. 1b). In this tissue, chondrocytes acquire osteoblast-like properties or transdifferentiate into osteogenic cells (Fig. 1b) and contribute directly to matrix mineralization^3,24,29–31^. There is no single factor that triggers the development of chondroid bone; rather, it is considered a complex developmental strategy during early ontogeny or/and under stress conditions to integrate proliferative properties of cartilage and structural properties of mineralized matrix allowing rapid growth of stable tissue^24^.

During the development of dermal bones, these elements expand in all directions through the deposition of new bone layers at both the external and internal cortices. Concurrently, resorption of the initially deposited bone layers produces a characteristic porous middle region, always secondary in origin. This process results in the formation of a diploë structure, which is commonly described in the dermal bones of amphibians and consists of compact external and internal cortices surrounding a spongy middle region^32–37^. The dominant primary tissue is a parallel-fibred matrix, with a pronounced contribution of Sharpey’s fibers and interwoven structural fibers^32,33,37^. Thus, existing studies of non-amniote tetrapod dermal bones present a fairly consistent view of dermal bone histology, indirectly indicating that these bones form primarily through direct intramembranous ossification and metaplastic ossification contributing to their formation, as inferred from the presence of structural fibers^32–35^.

To date, chondroid bone has been documented in fishes and amniotes, but remains unreported in non-amniote tetrapods, including both extant lissamphibians and extinct early tetrapods^4^. This raised the question of whether the absence of chondroid bone reflects a phylogenetic constraint, represents the inability of amphibians to produce this tissue, or is the result of undersampling.

We report the presence of previously unrecognized chondroid bone in the surangular of the lower jaw of the Permian temnospondyl *Sclerocephalus nobilis* (SMNS 97069), representing the first record of this tissue in a dermal bone outside Amniota. This finding provides new insights into the diversity of developmental processes involved in dermal bone formation among non-amniote tetrapods.

The temnospondyl amphibian *Sclerocephalus* is an iconic taxon from the Saar-Nahe Basin in southwestern Germany and represents one of the most comprehensively documented Paleozoic amphibian groups^38–44^. Adults could reach about 1.5 m in length and had an elongate trunk and laterally compressed tail^41^. Many specimens preserve lateral line sulci, indicating an aquatic lifestyle, with larvae using external gills and adults breathing with lungs^41,42,44^. The broad, flat skull and overall anatomy suggest that *Sclerocephalus* was a predator in freshwater lakes and rivers^44^. So far research on that taxon has focused on its morphology, taxonomy and ontogeny [^38–44^ and references therein], in contrast, the histology of *Sclerocephalus* remains poorly understood. The histology of a single postfrontal fragment was described by Witzmann^32^, confirming the typical diploë structure of dermal bones, characterized by moderate vascularization of the external cortex and the presence of cyclical growth marks in both cortices. A more comprehensive histological study is provided by Klein and Konietzko-Meier^44^ of a not fully grown individual of *Sclerocephalus nobilis* (SMNS 97069; Alsenz, Early Permian of Saar-Nahe Basin in southwestern Germany). Their work^44^ focuses on periosteal matrices and growth marks observed in several bones from a single individual, highlighting pronounced intraskeletal variability in the number of preserved growth marks. For the first time, the transition from larval to post-larval stages is also documented histologically, with larval tissue composed of an avascular faint parallel-fibred bone matrix^44^. The study further includes a preliminary analysis of growth marks of selected dermal elements of the skull and lower jaw, including the parietal, angular, and surangular, but detailed histological characterization of these bones was not provided.

We present here the histology of lower jaw bones from the same individual, with particular emphasis on the occurrence of chondroid bone in the dermal surangular, representing the first record of this tissue in amphibians. We further provide comparative histological data from the adjacent angular and articular elements of the same individual, to illustrate the variability of ossification onsets within this lower jaw.

## Results

**Fig. 2:**
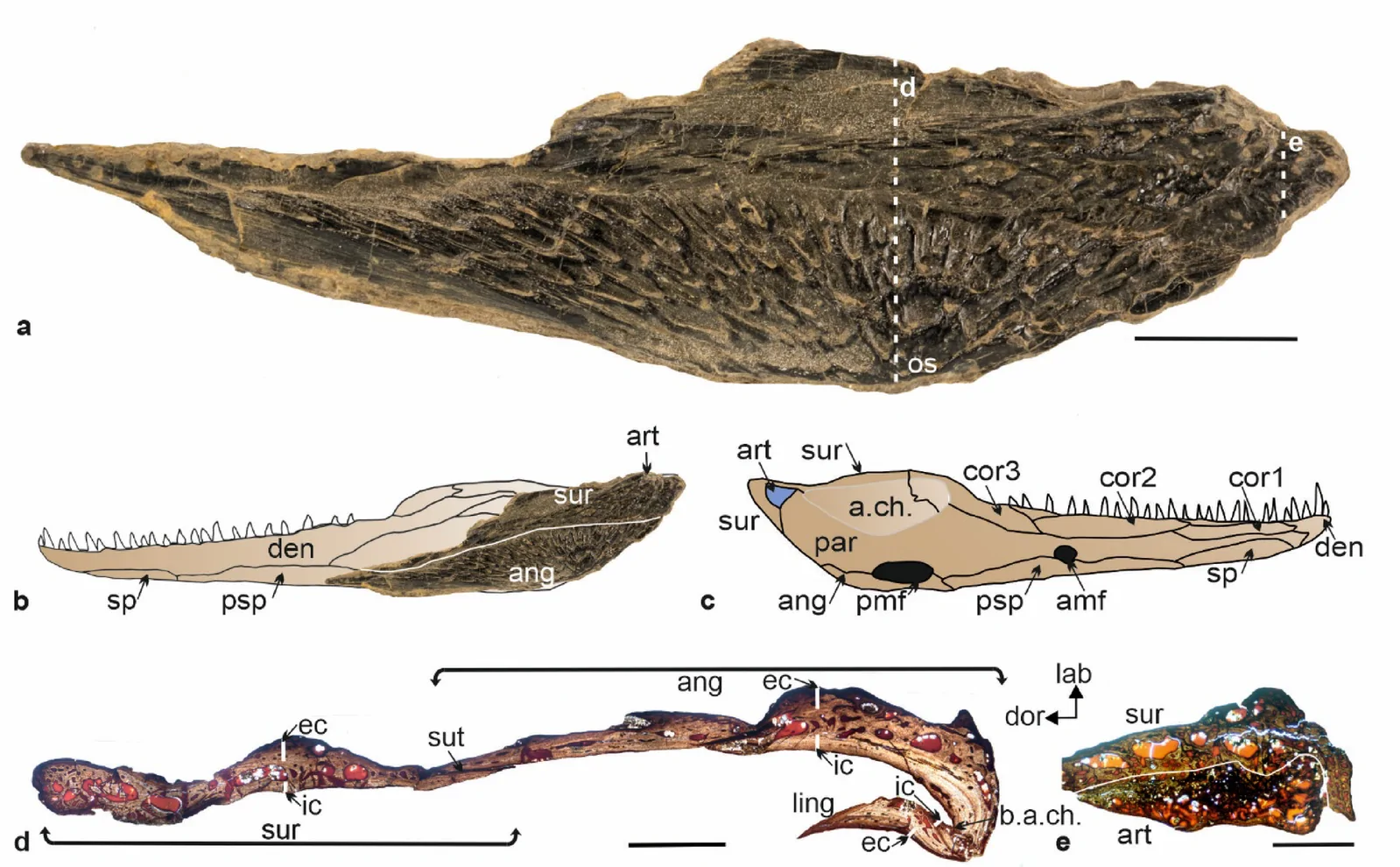
Studied material of *Sclerocephalus nobilis* (SMNS 97069) from the Lower Permian of Germany. (**a**) Fragment of left ramus of mandible in labial view, with angular and surangular preserved. The dashed lines mark the sampling planes. (**b**) Schematic of the lower jaw in labial view with marked position of the studied material (reconstruction after^40^). (**c**) The same jaw in lingual view, with marked position of the articular. (**d**) Overview image showing the vertical section of the angular (left) and surangular (right). (**e**) Overview of the section including articular and fragment of surangular. Scale bars equal: for (**a**) 10 mm, for (**d**) and (**e**) 2 mm. Abbreviations: a.ch. *adductor chamber*; amf *anterior Meckelian foramen*; ang *angular*; art *articular*; b.a.ch. *bottom of the adductor chamber*; cor1 *first coronoid*; cor2 *second coronoid*; cor3 *third coronoid*; den *dentary*; dor *dorsal*; ec *external cortex*; ic *internal cortex*; lab *labial*; ling *lingual portion of angular*; os *ossification center*; par *prearticular*; pmf *posterior Meckelian foramen*; psp *postsplenial*; sp *splenial*; sur *surangular; *sut* suture*.

The part of the lower jaw studied here (SMNS 97069) is composed of the angular, surangular, and articular (Fig. 2a). The angular and surangular form the main portion of the posterior wall of the mandible closing the adductor chamber (containing the jaw-closing muscles) on the labial side (Fig. 2a). The surangular contributes to the dorsal margin whereas the angular build the ventral portion and floor of the adductor chamber and extends slightly toward the lingual surface (Fig. 2b). The articular constitutes the most posterior bone of the mandible, lies medial to the surangular, and is visible only in dorsal view (Fig. 2c).

The specimen was sectioned at an easily reproduceable location that is perpendicular to the long axis of the jaw at the level of the ossification center of the angular (Fig. 2a). The ossification center is identified based on the characteristic ornamentation pattern on the bone surface, represented by an accumulation of honeycomb-like sculpturing (Fig. 2a). The section shows the angular ventrally and the surangular dorsally, separated by a suture (Fig. 2d). Both bones are dermal in origin, with ornamented labial surfaces forming the external cortex and the side facing the adductor chamber forming the internal cortex, separated by a spongy middle region (Fig. 2d). The second section done through the posterior part of the mandible shows the histology of the articular which is the only endochondral bone in the lower jaw and presents also part of the surangular (Fig. 2e).

**Fig. 3:**
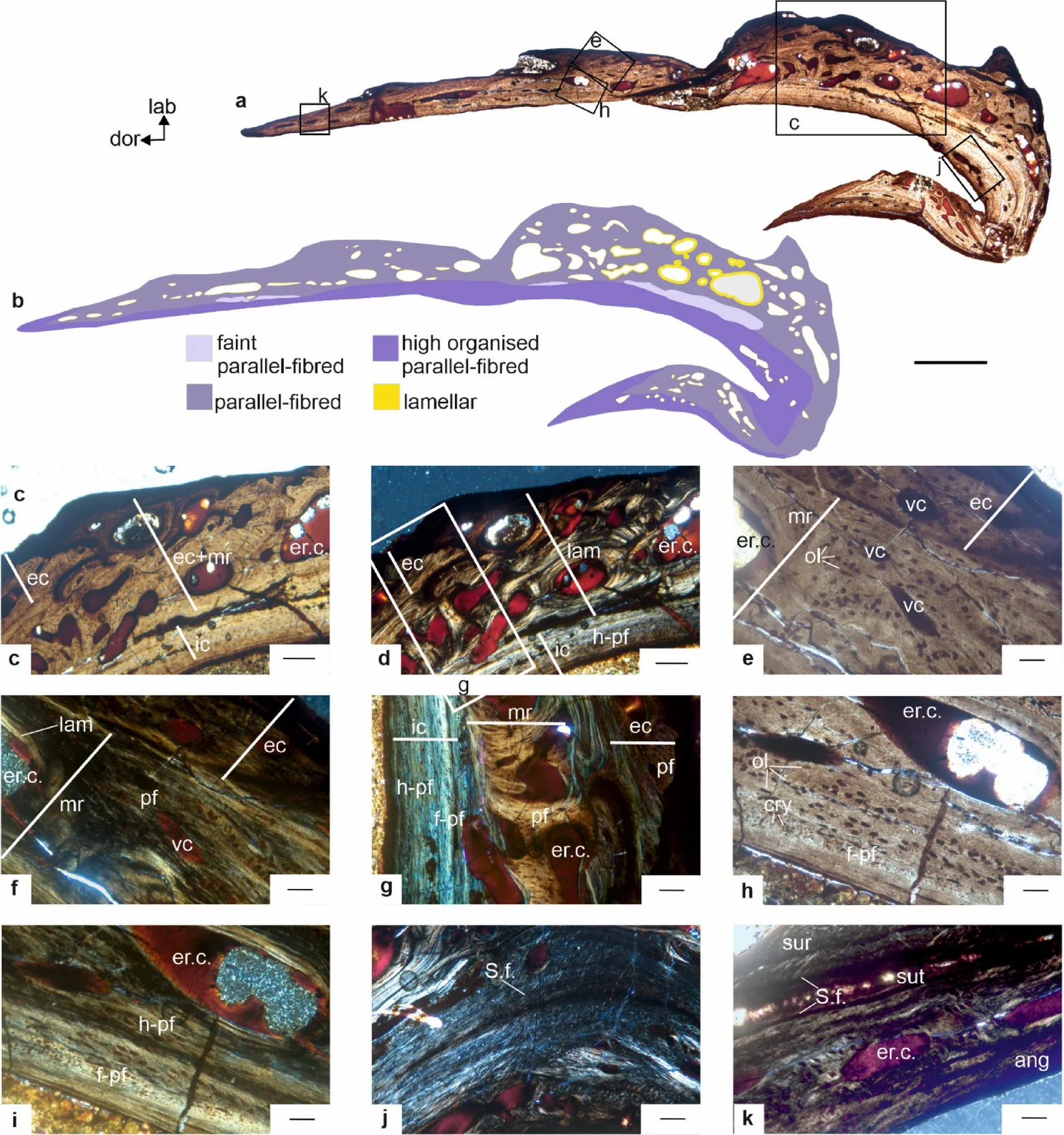
Osteohistological details of the angular of *Sclerocephalus nobilis* (SMNS 97069) from the Lower Permian of Germany. (**a**) The cross section of angular, image in plane-polarized light. (**b**) Idealized scheme of the angular displaying the distribution of different types of matrices. (**c**) Close-up of the angular from the region of the ossification center, image in plane-polarized light. (**d**) The same region in cross-polarized light; note the high degree of remodeling within the external cortex and middle region and the clear separation from the primary internal cortex. (**e**) Details of the external cortex in plane-polarized light. (**f**) The same region in cross-polarized light. (**g**) Close-up of (**d**) showing details of bone histology in circular polarized light. Note that the colors here do not have any meaning; the main advantage of this setting is the imaging of collagen fiber organization, making visible the separation between the two cortices and the middle region. (**h**) Close-up of the internal cortex with preserved remains of the avascular faint parallel-fibred bone. (**i**) The same region in cross-polarized light. (**j**) Details of the internal cortex at the bottom of the adductor chamber, hosting long Sharpey’s fibers, image in cross-polarized light. (**k**) The suture region between the angular and surangular with numerous short Sharpey’s fibers, image in cross-polarized light. Scale bars equal: for (**a**–**b**) 1 mm; (**c**–**f**) 250 µm; (**g**, **j**) 100 µm; (**h**–**i**, **k**) 50 µm. Abbreviations: cry *crystallite*; dor *dorsal*; ec *external cortex*; er.c. *erosion cavity*; f-pf *avascular faint parallel-fibred bone*; h-pf *highly organized parallel-fibred bone*; ic *internal cortex*; lam *lamellar*; lab *labial*; mr *middle region*; ol *osteocyte lacuna*; pf *parallel-fibred bone*; S.f. *Sharpey’s fiber*; sut *suture*; vc *vascular canal*.

**Angular**—The angular exhibits a typical diploë structure with visible separation into three layers: external cortex, middle region and internal cortex. The overall thickness of the bone decreases toward the surangular and the lingual tip (Fig. 3a-b). The boundary between the external cortex and the middle region is gradual, primarily distinguished by general cavity size, with larger erosion cavities present in the middle region (Fig. 3a-b). In the region of the ossification center, the entire external cortex merges with the middle region into a structurally uniform, highly remodeled layer (Fig. 3c-d). In contrast, the boundary between the middle region and the internal cortex is always distinct, marked by a change in matrix organization and by the absence of primary or secondary porosity in the internal cortex (Fig. 3a-d).

The external cortex of the angular is moderately vascularized, containing secondary enlarged vascular canals (Fig. 3e-f). The primary matrix shows a distinct birefringence pattern, appearing as a bright, subtly banded optical signal characteristic for parallel-fibred bone indicating a well-developed collagen fiber network (Fig. 3e-g). The erosion cavities are lined with secondary lamellar bone (Fig. 3f). Osteocyte lacunae are scattered and round in the parallel-fibred region, but flattened and arranged in rows within the lamellar bone (Fig. 3e).

Within the middle region, the size of erosion cavities gradually decreases from large erosion cavities in the ossification center to secondary enlarged vascular canals dorsally and toward the lingual tip (Fig. 3a-b). The secondary originating middle region contains numerous areas with preserved primary matrix. It exhibits the same characteristics as the external cortex and transitions smoothly between these two regions (Fig. 3e-g). The erosion cavities are lined by secondary lamellar bone. Along the border between the middle region and the internal cortex, and locally within the internal cortex, small islands of faint parallel-fibred matrix with only a weak optical reaction suggesting the lower mineralization of matrix (for details see 44), (Fig. 3g-i). Additionally, in plane-polarized light, these fragments are lighter in color than the surrounding bone and are rich in iron crystallites (Fig. 3h). This distinctive type of matrix, discussed in detail by Klein and Konietzko-Meier^44^, is interpreted as avascular faint parallel-fibred bone representing the histology of an earlier ontogenetic stage, i.e. the larval phase of this individual.

The internal cortex is composed of avascular high organized parallel-fibred grading into lamellar bone (Fig. 3h-i). Osteocyte lacunae in inner cortex are flat and arranged in rows (Fig. 3h). The internal cortex in the most ventral part, constructing the bottom of the muscle chamber, hosts long, numerous Sharpey’s fibers (Fig. 3j). Short Sharpey’s fibers are as well visible in the region of the suture with the surangular (Fig. 3k).

**Fig. 4:**
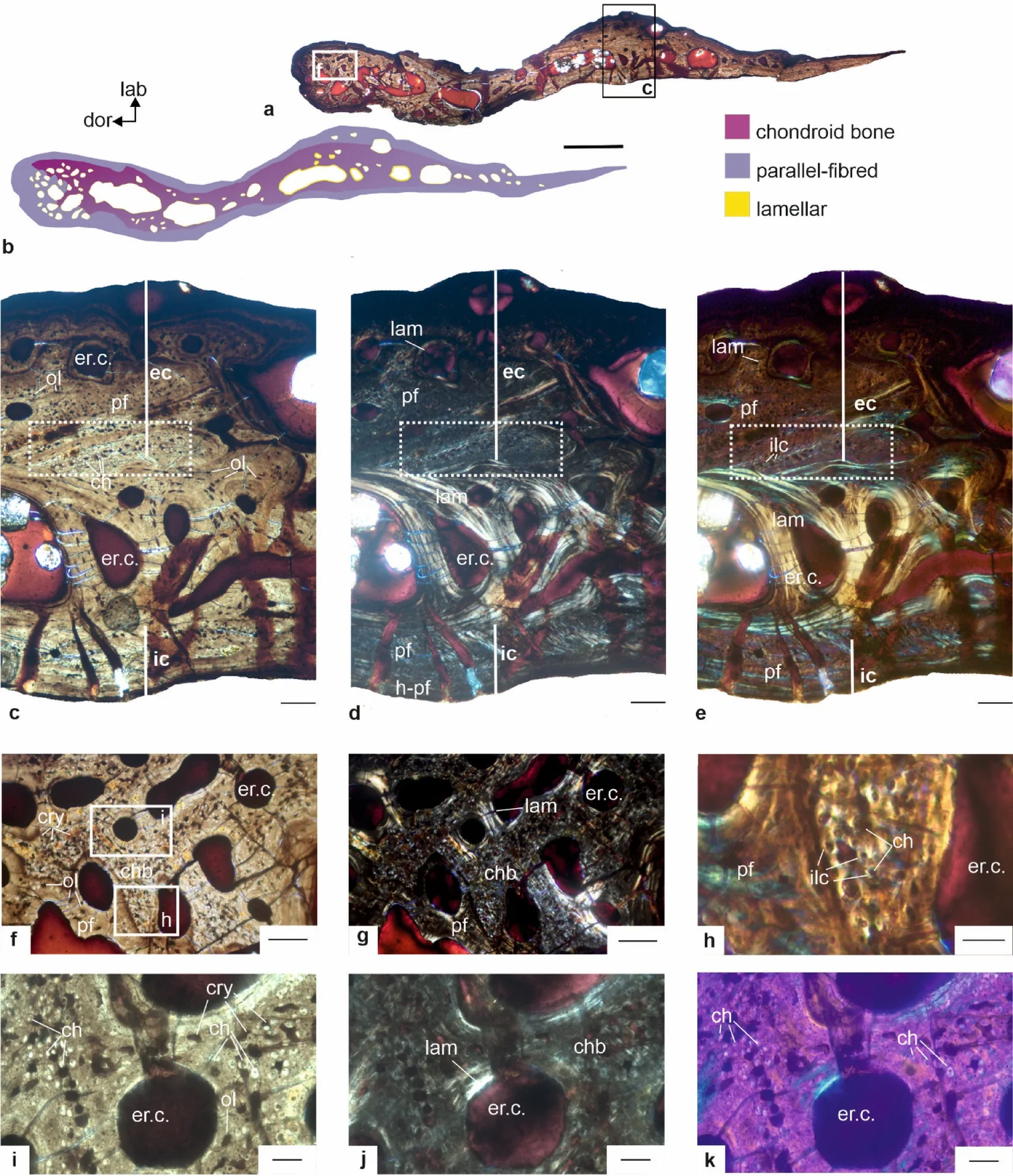
Osteohistological details of the surangular of *Sclerocephalus nobilis* (SMNS 97069) from the Lower Permian of Germany. (**a**) Cross-section of the surangular, image in plane-polarized light. (**b**) Idealized scheme of the surangular displaying the distribution of different types of matrices. (**c**) Close-up of the surangular from the region of the ossification center, image in plane-polarized light. Note the small areas of chondroid bone close to the middle region outlined by a dashed rectangle. (**d**) The same region in cross-polarized light. (**e**) The same region in circular polarized light. Note that the colors here do not have any meaning. Interwoven loop complexes are visible in areas with chondroid bone. (**f**) Close-up of the dorsal portion of the bone, with chondroid bone visible between the erosion cavities. (**g**) The same region in plane-polarized light. (**h**) Detail of the chondroid bone showing collagen fibers arranged into interwoven loop complexes and embedded chondrocyte-like cells. Note the distinct matrix architecture compared with the adjacent parallel-fibred bone visible in the same image, image in circular polarized light. (**i**) Close-up of (**f**) with visible whitish, oval chondrocyte-like cells. (**j**) The same region in plane-polarized light and with a lambda filter (**k**). Scale bars: (**a**–**b**) 1 mm; (**c**–**g**) 100 µm; (**h**–**k**) 25 µm. Abbreviations: ch *chondrocyte lacuna*; chb *chondroid bone*; cry *crystallite*; dor *dorsal*; ec *external cortex*; er.c. *erosion cavity*; f-pf *avascular faint parallel-fibred bone*; h-pf *highly organized parallel-fibred bone*; ic *internal cortex*; ilc *interwoven loop complex*; lam *lamellar*; lab *labial*; ol *osteocyte lacuna*; pf *parallel-fibred bone*; S.f. *Sharpey’s fiber*.

**Surangular**—In the surangular, the diploë structure is best preserved in the ventral portion of the bone (Fig. 4a-b). The external cortex hosts small erosion cavities lined with lamellar bone (Fig. 4c-d). The middle region is highly remodeled and characterized by a layer of large erosion cavities. Unlike in the angular, it lacks a distinct boundary and gradually transform into the avascular internal cortex. In both cortices, the matrix is parallel-fibred, but only indistinct bundles of collagen fibers are visible (Fig. 4c-e). However, no change of color in plane polarized light compared to the surrounding tissue is observed, and only a moderate amount of iron crystallites is present, distinguishing it from the avascular faint parallel-fibred matrix observed in the angular (Fig. 4c-e vs. Figure 3h). Only the outermost layer of the inner cortex and the secondary bone of the erosion cavities present the middle region shows, highly organized parallel-fibred and lamellar matrix, respectively (Fig. 4c-e). Generally, the overall organization of the matrices is lower as in the angular (Fig. 4h vs. Figure 3g). The osteocyte lacunae are round and scattered in parallel-fibred parts and flat and aligned in the higher organized matrices (Fig. 4i).

Toward the dorsal margin the diploë structure, with separation into three layers, disappears completely, and the bone becomes structurally homogeneous, with numerous small erosion cavities distributed throughout the cross-section (Fig. 4a-b). In this region, the outermost cortex of the bone consists of a thin layer of avascular high organized parallel-fibred bone (Fig. 4a). Additionally, islands of parallel-fibred bone extend into the inner part of the bone between the numerous cavities, which are themselves lined with lamellar bone (Fig. 4f-g). The most striking character in the surangular is the matrix present between the cavities in the dorsal part. The collagen fibers here form characteristic loop structures (Fig. 4h), the interwoven loop complexes—ILCs as introduced by Kalita et al.^37^. The ILC refers to a distinctive matrix organization characterized by an interconnected network of loop-like and interwoven structures formed by collagen fiber bundles. This pattern is particularly evident under circular polarized light, where the complex appears as a mesh-like arrangement of intertwined fibers (Fig. 4h). In the surangular this matrix is interspersed by a high concentration of iron-crystallites (Fig. 4f, i). Some of the iron crystallites resemble the shape of osteocyte lacunae; however, osteocyte lacunae can be distinguished by the presence of canaliculi and their regular shape, whereas the crystallites are typically more cubic, angular, and irregular in outline (Fig. 4i). Osteocyte lacunae are absent within the ILCs matrix.

The ILCs matrix contains numerous whitish, oval structures visible in plane-polarized light. These structures occur either individually or in clusters of two elements (Fig. 4f, i–k). This structure is characteristic of chondrocytes^4,45^ and interpreted by us as chondrocyte lacunae. These lacunae are larger than the osteocyte lacunae observed in the surrounding parallel-fibred matrix, lack the extensive canalicular network typical of osteocytes, and are distinct from the iron crystallites by color and shape (Fig. 4i-k). The crystallites are irregular in shape and usually brown to dark brown, whereas the chondrocyte lacunae are dominantly regularly oval and always whitish. They are predominantly located at the dorsal-most tip of the surangular embedded within a matrix composed of ILC (Fig. 4f-g), but also appear locally in the region of ossification center of the surangular, accompanied with ILC and enclosed by parallel-fibred matrix (Fig. 4c-e). The presence of numerous chondrocyte lacunae, the absence or extreme scarcity of osteocyte lacunae, and the matrix characteristic that is clearly distinct from the surrounding parallel-fibred bone support the identification of these areas as chondroid bone (Fig. 4h).

**Fig. 5:**
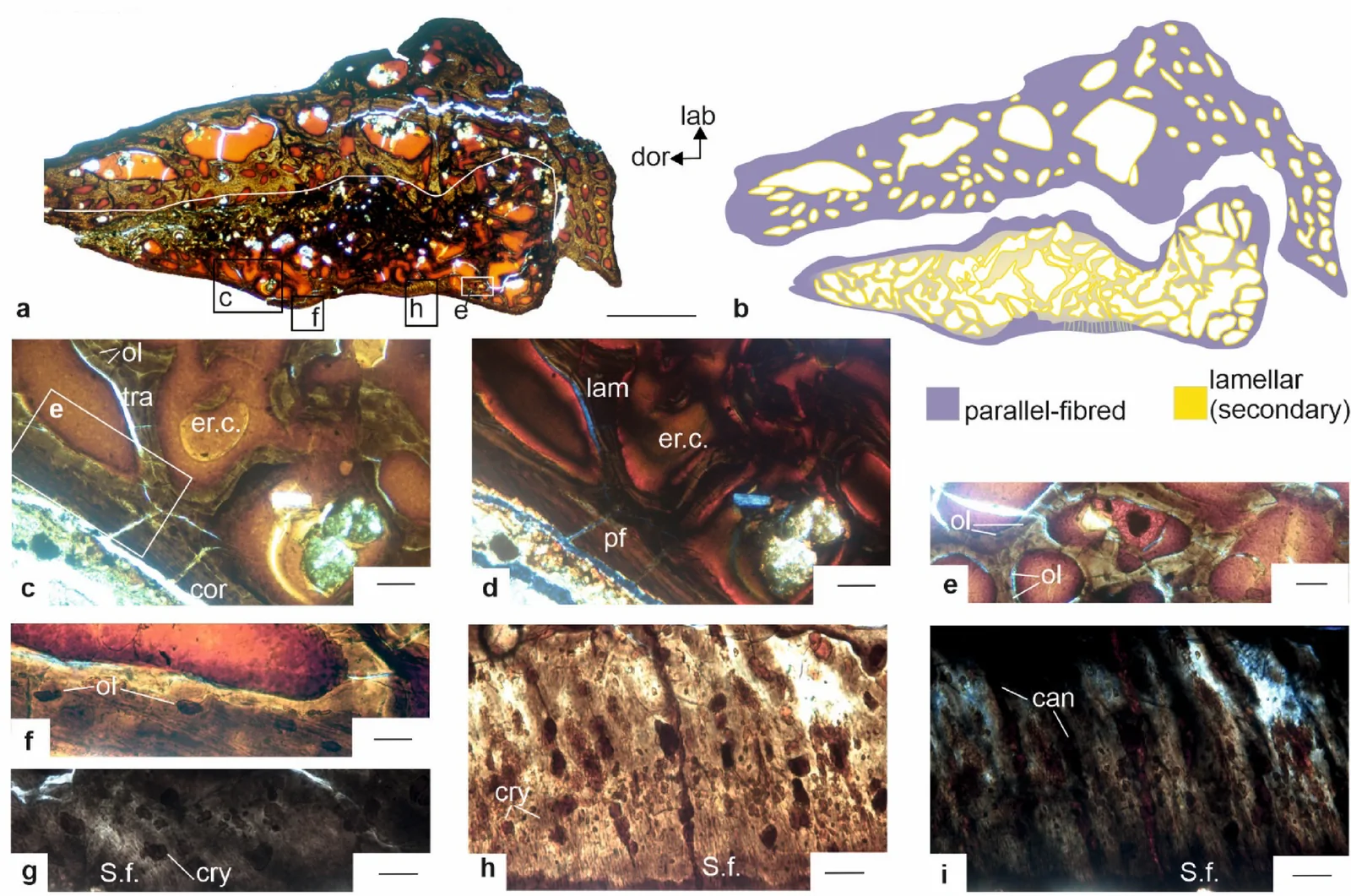
Osteohistological details of the articular of *Sclerocephalus nobilis* (SMNS 97069) from the Lower Permian of Germany. (**a**) Cross-section of the articular, image in plane-polarized light. From the labial side, the articular is covered by a fragment of the surangular. The white line marks the probable course of the suture between these two bones. (**b**) Idealized scheme of the articular displaying the distribution of different types of matrices. The distance between the surangular and the articular is here deliberately increased to show details of the articular. (**c**) Close-up of the articular periosteal cortex and fragment of the secondary trabecular part, image in plane-polarized light. (**d**) The same region in cross-polarized light. (**e**) Close-up of the trabecular bone, with visible osteocyte lacunae arranged in rows, image in plane-polarized light. (**f**) Close-up of the periosteal cortex, with visible osteocyte lacunae, image in plane-polarized light. (**g**) Gentle Sharpey’s fibres visible in the cortex, image in cross-polarized light. (**h**) Sharpey fiber bone and numerous short fibers in the outermost cortex of periosteal bone. (**i**) The same region in plane-polarized light. Note the thick column-like structures representing remains of thick Sharpey’s fibers. Scale bars: (**a**–**b**) 1 mm; (**c**–**d**) 50 µm; (**e**) 100 µm; (**f**–**i**) 25 µm. Abbreviations: can *canals*; cor *periosteal cortex*; cry *crystallite*; dor *dorsal*; er.c. *erosion cavity*; lam *lamellar*; lab *labial*; ol *osteocyte lacuna*; pf *parallel-fibred bone*; S.f. *Sharpey’s fiber*; tra *trabeculae*.

**Articular—**The articular is the only endochondral bone of the lower jaw. The section of the articular is partially collapsed. However, its microstructure and histology are very distinct from that of the dermal bones (Fig. 5a-b). The preserved part of the articular shows a trabecular structure surrounded by a thin layer of cortex. The trabecular part of the articular is mostly secondary in nature, composed of endosteal lamellar bone (Fig. 5a, c-d), containing spindle-shaped and in rows aligned osteocyte lacunae (Fig. 5e). No cartilage remnants are identified between the trabeculae.

Along the margin to the prearticular (not preserved in the studied sample), the articular shows a thin layer of avascular matrix (Fig. 5c-d, f). This layer exhibits mixed histological properties, predominantly showing a parallel-fibred matrix with visible collagen fibers and a few osteocyte lacunae arranged in rows (Fig. 5c-d, f). Short Sharpey’s fibers are present throughout the preserved cortex (Fig. 5g). The cortical structure of the *Sclerocephalus* articular is characterized by local areas penetrated by numerous thick, prominent radial struts, which most likely represent canals left by massive Sharpey’s fibers extending into the bone from the external surface (Fig. 5h-i). This pattern is classified as Sharpey fiber bone, in which the fibers act as a scaffold during the initial ossification process^46,47^. In addition to bundles of very massive fibers, numerous fine, short fibers are present in the outermost part of cortex (Fig. 5h-i).

## Discussion

The most striking structures in the lower jaw of *Sclerocephalus nobilis* (SMNS 97069) from Alsenz are chondrocyte lacunae embedded within interwoven loop complexes (ILCs). The latter represent the primary collagen scaffold of the intramembranous bone, which is subsequently infilled by additional fibers, giving rise to the final matrix structure^37^. The combination of cartilaginous (chondrocytes) and osseous (ILCs) features is consistent with chondroid bone^2^. So far, among Temnospondyli, cartilage remnants associated with the skull and mandible bones have been reported only in endochondral bones (exoccipital, quadrate, and articular), where they occur as islands of calcified cartilage surrounded by lamellar trabeculae^49,50^. However, this combination does not indicate chondroid bone, as chondrocytes are not embedded within the bony matrix; instead, bone and cartilage form two clearly separated domains^48,49^. Thus, the presence of chondroid bone in the surangular of *Sclerocephalus* represents the first record of this tissue among non-amniote tetrapods and indicates possible multiple developmental pathways of bone formation on the histological level within one specimen (Fig. 1).

Chondroid tissue has been documented during early ontogeny in craniofacial regions of fishes and amniotes undergoing rapid growth and/or mechanical stimulation^2,4,22^. Specimen SMNS 97069 is suggested to be, based on its morphology, an individual of a late juvenile stage^41,42,44,50^. The presence of avascular faint parallel-fibred bone in the long bones^44^ and angular, as well as the occurrence of chondroid bone of the surangular, further supports an early ontogenetic stage for this individual.

The juvenile stage of the specimen is essential for understanding the function of chondroid bone in the surangular. The surangular occupies a biomechanically strategic position in the adductor chamber. It serves as an attachment surface for the muscle *adductor mandibulae externus* and participates in transmitting forces generated by the jaw adductor musculature^51^. The structure is presumably exposed to repetitive biomechanical loading during feeding. Consequently, early ontogenetic development and accelerated growth would be critical to secure functional articulation with the skull and maintain feeding efficiency. This suggests that chondroid bone is locally promoted in the surangular, as in the jaw articulation of fishes^5,52^ and allows to achieve functionality earlier than neighboring bones that develop via intramembranous ossification. Together, these observations support the interpretation that chondroid bone in the surangular of *Sclerocephalus*, is a non-pathological tissue during early ontogeny that may serve both developmental and functional roles, promoting rapid growth and providing support in regions subjected to high stress during feeding and movement of jaw elements. A pathological origin of the chondroid bone in the surangular, such as a chondroid tumor, can be excluded. Chondroid tumors are typically histologically distinct from physiological chondroid bone, showing different matrix and vascularization accompanied by morphological abnormalities [53 and references therein].

Interestingly, chondroid bone was not identified in any of the endochondral bones of the studied individual^44^. Whether chondroid bone is specific only to the surangular of this specimen, present in other individuals of *Sclerocephalus*, or maybe more generally characteristic for larval ontogenetic stages of early tetrapods, requires further study. Histological studies of the lower jaw and skull in early tetrapods are extremely rare; a detailed histological description of the mandible has so far been provided only for *Metoposaurus*, a stereospondyl taxon from the Late Triassic^48,49^. In this taxon, the surangular and angular are identical in terms of histological organization and compactness^50^. The main differences among individual bones within a single skull or mandible of metoposaurs are primarily expressed in bone porosity and in the relative proportions of the three principal layers of dermal bone, rather than in matrix composition^48,49,54^. However, it should be noted that the studied specimens of *Metoposaurus* represent adult individuals^49^, which likely influenced their histology and therefore may limit their value as a direct comparative model for the juvenile *Sclerocephalus.* Thus, to better clarify the extent of chondroid bone occurrence in early tetrapods, the examination of both endochondral and dermal bones in very early juvenile stages of other temnospondyls will be crucial for assessing variability in ossification patterns across taxa.

 The presence of chondroid tissue in a dermal bone of *Sclerocephalus* suggests that chondroid bone may have been more widespread among temnospondyls and possibly early tetrapods during early ontogeny, and that its occurrence may have been overlooked due to preservational biases and limited histological sampling. The apparent absence of chondroid bone among lissamphibians may result from undersampling and/or misidentification in conventional histological sections as well. Among Lissamphibia, several cartilage-related structures have been described for long bones, such as globuli ossei^55,56^ and the Kastschenko line [^57,58^ and references therein]. However, neither corresponds to the definition of chondroid bone, which is characterized by the presence of chondrocytes embedded within a mineralized bone matrix^2^. On other hand, Kondo et al.^59^, in their study on the early ontogeny of anuran long bones, illustrate chondrocytes surrounded by bone matrix [^59^: Fig. 2b], but do not refer to this tissue as chondroid bone. This suggests that at least type I chondroid bone associated with endochondral ossification may be under-recognized in the literature, and potentially is present more broadly during bone development than currently documented.

Apart from the undesampling, the phylogenetic factor, however, cannot be excluded, especially for dermal structures, as surangular. Although Lissamphibia are phylogenetically closely related to Temnospondyli [^60–62^ and references therein], they exhibit extensive cranial modification and simplification (reduction of elements) relative to early tetrapods, with numerous dermal bones reduced, fused, or lost due to altered developmental trajectories and functional reorganization of the skull and mandible^62,63^. Consequently, the complex multi-element mandible of early tetrapods, including bones such as the surangular, is not retained as distinct elements in living amphibians^62^ and can limit the potential for creation of the chondroid bone.

**Fig. 6:**
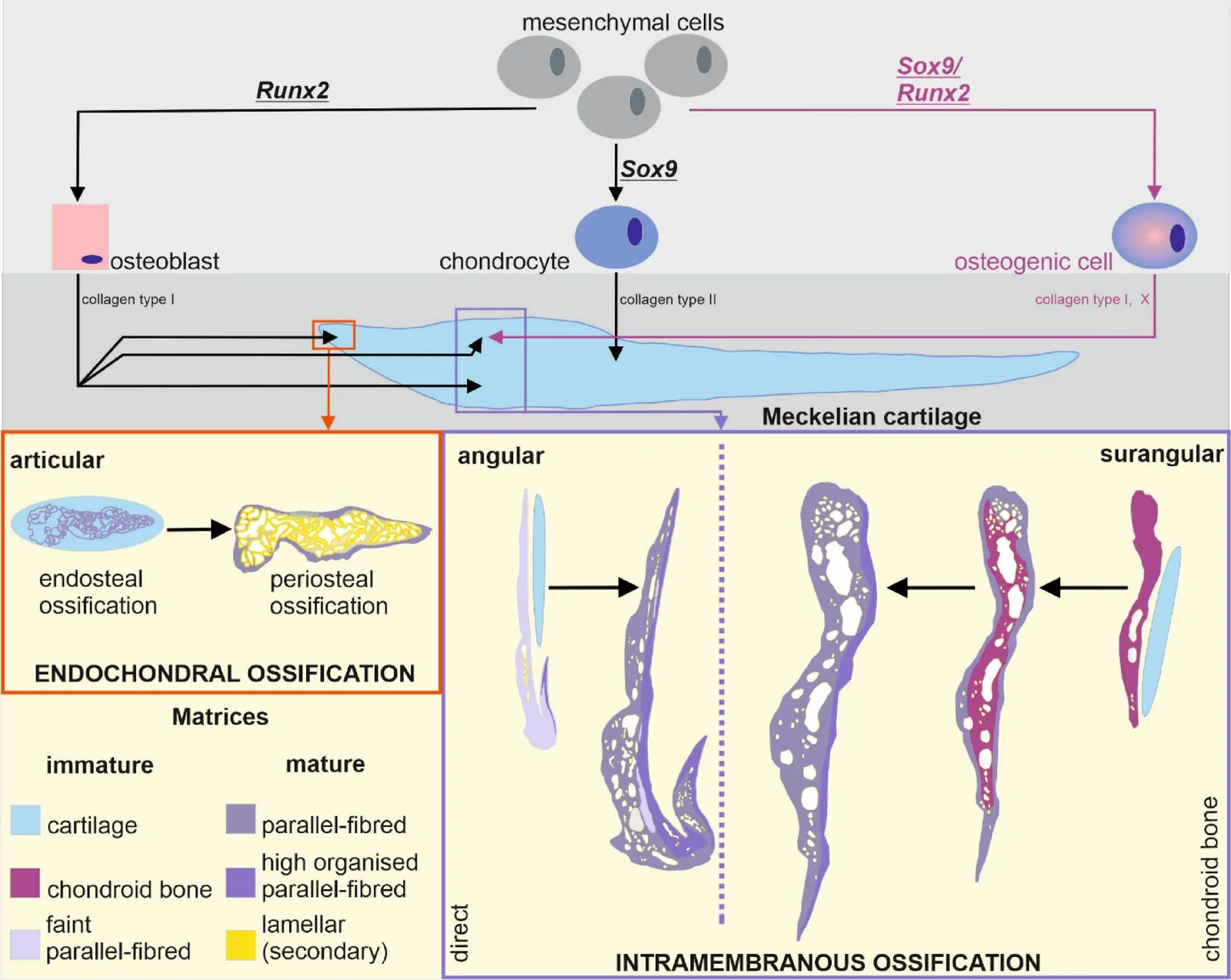
Simplified scheme illustrating the different ossification pathways in the lower jaw of *Sclerocephalus nobilis* (SMNS 97069) from the Lower Permian of Germany. Lower jaw formation begins with the condensation of mesenchymal cells, which differentiate into chondroblasts under the control of SOX9, giving rise to Meckelian cartilage. In endochondral ossification (articular), osteogenesis is subsequently initiated by a later population of RUNX2-controlled mesenchymal progenitors that differentiate into osteoblasts and establish the first endochondral bone within the cartilage, followed by periosteal bone deposition on its surface (red frame). Dermal ossification (violet frame), which occurs around the cartilaginous template, involves the direct differentiation of mesenchymal cells into osteoblasts under the control of RUNX2, producing a larval avascular, faint parallel-fibred bone matrix (angular). Alternatively, mesenchymal cells may simultaneously activate osteogenic and chondrogenic programs (RUNX2/SOX9), resulting in the formation of a second type of immature matrix, chondroid bone (surangular). As ontogeny proceeds, both immature matrices are followed by the deposition of mature parallel-fibred bone. The immature matrices may subsequently undergo secondary remodeling into secondary lamellar bone, which forms the middle region of the dermal bones. For further explanation, see the text. The concept of this scheme is based on^24,44^.

The angular of *Sclerocephalus* shows the typical framework of diploë bone with two cortices and the middle region. Developmentally, the small inclusions in the middle region of avascular faint parallel-fibred matrix in the angular are important, as they represent remnants of larval bone^44^. The larval matrix of the angular differs markedly from that of the surangular. The faint avascular parallel-fibred matrix is characteristic of early ontogeny only^44^, whereas the ILCs matrix represents the first type of matrix deposited during the growth of dermal bones, as suggested for *Metoposaurus*, and it also occurs in adult animals^37^. The ILCs in the *Sclerocephalus* specimen analyzed here indeed represent a matrix present during early ontogenetic stages, and are considered to be equivalent to the larval stage. It is important to note that the larval tissue in the angular does not correspond to a later developmental stage of the ILC matrix, or vice versa. Instead, these two larval tissue types (avascular faint parallel-fibred and ILCs characteristic for chondroid bone) observed in the dermal bones exist parallel, reflecting different ossification pathways: direct intramembranous ossification in the angular and ossification via chondroid bone in the surangular (Fig. 6).

The third type of ossification is represented in the articular bone as a remnant of the Meckelian cartilage (Fig. 6). The articular of *Sclerocephalus* exhibits a structure closely similar to the histology of the articular of *Metoposaurus*, characterized by a highly trabecular architecture of secondary origin and a thin primary compact cortex^49^. In *Metoposaurus*, numerous remnants of calcified cartilage are preserved between the trabeculae; in contrast, no cartilage is observed between the trabeculae in *Sclerocephalus*. In the articular, the dense Sharpey’s fibers likely function as a sutural connection to the prearticular or may directly represent the articular facet with the skull. Unfortunately, due to preservation of the specimen, it is not possible to confirm whether similar structures are present in the suture between the surangular and articular.

## Conclusion

Up to date most histological studies of early tetrapods, largely based on temnospondyls, indicate only two modes of ossification in the lower jaw (Fig. 6), with dermal bones ossifying directly from mesenchyme, and the articular forming endochondrally from Meckelian cartilage [^49^ and references therein]. For the specimen examined here, endochondral bones appear to follow a conserved ossification pattern (see^44^ for long bones histology), with the cortex reflecting periosteal deposition, whereas dermal elements exhibit greater heterogeneity during juvenile stages. The discovery of chondroid bone within a single jaw element of *Sclerocephalus nobilis* from Alsenz (SMNS 97069) suggests that dermal bones may form through an additional ossification pathway involving chondroid bone, besides direct intramembranous and endochondral ossification (Fig. 6). This variability may be linked to the species’ high morphological plasticity [^43^ and references therein], but also to the early ontogenetic stage of this peculiar specimen.

The occurrence of chondroid bone in this Early Permian *Sclerocephalus* represents the only confirmed record of this tissue in Amphibia and the earliest known occurrence in the vertebrate fossil record to date. Its presence in a taxon exhibiting a mosaic of plesiomorphic “fish-like” and derived early tetrapod features [^43^ and references therein] highlights the high developmental plasticity of the early tetrapod skeleton and fills a phylogenetic gap between previously documented occurrences of this tissue in fishes and amniotes. The presence of chondroid bone in the surangular underscores the potential occurrence of transitional tissue types in early tetrapods and suggests that such structures may have been more widespread than currently recognized, but underreported due to preservational biases and limited histological sampling. This, in turn, raises the question of whether the apparent absence of similar tissues in Lissamphibia reflects an evolutionary loss or instead a sampling gap resulting from limited histological data and taphonomic constraints.

## Material and methods

A fragment of the left lower jaw from the *Sclerocephalus nobilis* specimen (SMNS 97,069; Alsenz locality, Klauswald facies of the Kappeln black shale; M9-K; skull length 80 mm; after^43^) was studied. It includes the angular, surangular, and articular bones (Fig. 2). The specimen was sectioned perpendicular to the long axis of the jaw at the level of the ossification center of the angular (Fig. 2a). A second cut was made through the articular (Fig. 2a). In lateral view, the bone is morphologically covered by the surangular and is visible only in the CT image.

The fragment was additionally micro-CT scanned before cutting to precisely locate the sectioning planes. Scanning was performed using a v|tome|x s scanner manufactured by GE phoenix|X-ray [Wunstorf, Germany, housed at the Institute of Organismic Biology, Section Paleontology, University of Bonn, Germany (BIOB-V).

Thin sections were prepared following the procedures described by Klein and Sander^64^ at BIOB-V. Osteohistological analysis was carried out using a Leica DM LP polarizing light microscope equipped with a Leica K5C camera and fitted with a circular polarization setting, following Kalita et al.^37^. Histological terminology follows Francillon-Vieillot et al.^55^ and Buffrénil et al.^65^. Please note, that in this paper we want to focus on the chondroid bone and variability of matrices, the growth marks analyses are part of a separate paper^44^.

## Data Availability

All data generated or analyzed during this study are included in this article, the thin sections are housed and available on request in SMNS.
